# Comparative Assessment of Lipid Peroxidase in Oral Cancer and Oral Potentially Malignant Disorders

**DOI:** 10.7759/cureus.66474

**Published:** 2024-08-08

**Authors:** Dhanya M, Jayanth Kumar Vadivel, Umamaheswari T.N, Selvaraj Jayaraman

**Affiliations:** 1 Oral Medicine and Radiology, Saveetha Dental College, Saveetha Institute of Medical and Technical Sciences, Chennai, IND; 2 Centre of Molecular Medicine and Diagnostics (COMManD) Department of Biochemistry, Saveetha Dental College, Saveetha Institute of Medical and Technical Sciences, Chennai, IND

**Keywords:** malignant risk, oxidative stress, lipid peroxidase, oral potentially malignant disorders (opmd), oral cancers

## Abstract

Introduction

Oral potentially malignant disorders (OPMD) are abnormally altered tissues that could potentially develop into oral cancer. From the literature, it is understandable that not all OPMDs develop into oral cancer. Hence, it is essential to identify the high-risk lesions that are more likely to develop into oral cancer. Lipid peroxidase (LPO) is a byproduct of phospholipid metabolism, and its levels are an oxidative stress marker that can probably help us predict the onset of cancer in OPMDS. This study aimed to assess the levels of LPO in OPMD, oral cancer, and normal patients.

Materials and methods

The sample size estimated was 15 per group. There were four groups in total. The estimation was done with the Abbkine LPO enzyme-linked immunosorption assay (ELISA) kit (Atlanta, Georgia, USA). An enzyme-substrate reaction was carried out, and the degree of the color change was measured using a microplate reader. The values were tabulated, and statistics were carried out using Statistical Package for Social Sciences (SPSS) version 26.0 (IBM Corp., Armonk, NY, USA). Both descriptive and inferential statistics were carried out.

Results

LPO levels (nmol/L) in each of the four groups were as follows: Group 1 (oral cancer): 171.86±78.86, Group 2 (controls): 71.66±28.36, Group 3: (oral leukoplakia): 127.50±103.53, and Group 4 (oral submucous fibrosis and oral lichen planus): 100.39±41.06. The results, when compared, were statistically significant (P< 0.05).

Discussion

From the above results, it is understandable that oral cancer patients experience increased oxidative stress compared to the OPMD group. The current study concluded that the obtained results showed differences in LPO levels, suggesting LPO could be used as a marker and screening tool to assess the rate and severity of cellular damage in patients with oral potentially malignant disorders.

## Introduction

Oral potentially malignant disorders (OPMD) are abnormally altered tissues that could potentially develop into oral cancer. Some of the most common OPMD are oral submucous fibrosis, oral leukoplakia, erythroplakia, and oral lichen planus [1]. The etiology for developing the above lesions includes smoking, smokeless tobacco chewing, and areca nut chewing. The use of alcohol does not cause any of the above OPMD but can be a potentiating factor by dehydrating the mucosa, allowing for the easier penetration of the above carcinogens [2]. The overall global prevalence of OPMD was 4.47% [3]. OPMD is a prelude for the formation of oral cancers, which initially present a visible change in the oral mucosa, manifesting as a white or red patch [4]. Depending on the grade of dysplasia and clinical subtype, the overall malignant transformation rate of OPMD was 7.9%, out of which oral lichen planus was 1.4%, oral submucous fibrosis was 5.2% and that of leukoplakia was observed to be 9.5% [5].

In the development of carcinogenesis, oxidative stress is caused by an imbalance of free radicals and the body’s antioxidant level, which can cause cellular damage. Reactive oxygen species (ROS) are a subtype of free radicals that are derived from oxygen such as superoxides. These are produced during the normal metabolic process. Among all cell organelles, mitochondria are the main generators of ROS [6,7]. The ROS is produced in response to the carcinogens present. When there is an imbalance between the formation of reactive oxygen species and antioxidant levels, it causes oxidative stress in cellular components, which impacts the DNA, proteins, and lipids of the cells [8]. According to the World Health Organisation (WHO), biomarkers are products or substances that play a vital role in assessing the incidence, severity, or outcome of the disease [9].

Lipid peroxidase (LPO) is an oxidative stress marker that plays a crucial role in the cellular mechanisms of OPMD and oral cancers [10]. Lipid peroxidation is a process brought on by ROS, indicating a sign of severe cellular damage [11]. Many human disorders have been linked to lipid peroxidation caused by free radicals. To test lipid peroxidation products as potential biomarkers and to evaluate the status of oxidative stress, different techniques have been employed [12]. The most common method is to assess the aldehyde product by utilizing its ability to react with thiobarbituric acid [13]. ROS from tobacco is an external source that causes oxidative stress (OS). Polynuclear aromatic hydrocarbons (PAH) and nitrosamines found in tobacco products promote the generation of ROS and free radicals (ROS), which have a pathognomonic role in the multistep carcinogenesis process. They start mutagenesis processes by damaging DNA, which eventually results in the deterioration of biological components. The peroxidation of polyunsaturated fatty acids in membrane lipids is the primary target of ROS and can be used as a biomarker for the early detection and monitoring of oral potentially malignant disorders. However, LPO is not a specific marker for oral cancer and is found to be elevated in chronic periodontitis and other inflammatory disorders as well [11,14-16].

The core aim of the study was to assess the levels of LPO in oral cancer and oral potentially malignant disorders. The primary objective of the study was to compare the levels of LPO in controls with that of the experimental groups (oral cancer, oral submucous fibrosis, and oral leukoplakia) and the secondary objective was to compare the levels of LPO between different types of OPMD lesions.

## Materials and methods

The observational clinical study was conducted among patients reporting to the Department of Oral Medicine and Radiology. The study received the approval of the Institutional Ethics Committee of Saveetha Dental College vide Ref. No.: IHEC/SDC/OMED-2103/23/212. The inclusion and exclusion criteria are mentioned in Table 1.

**Table 1:** Inclusion and exclusion criteria of the participants in the study

Inclusion Criteria	Exclusion Criteria
Patients in the 18-60 age group.	Patients with systemic diseases like diabetes, hypertension, anemia, and a previous history of malignancy could be confounding variables and interfere with the biopsy procedure.
Patients with clinically confirmed diagnoses of oral cancer, oral leukoplakia, and oral submucous fibrosis	Patients whose biopsy reports were inconclusive or nonconsistent with the clinical diagnosis
Patients who had histopathologically confirmed diagnoses of oral cancer, oral leukoplakia, and oral submucous fibrosis	Patients who did not consent to participate in the study

**Figure 1:**
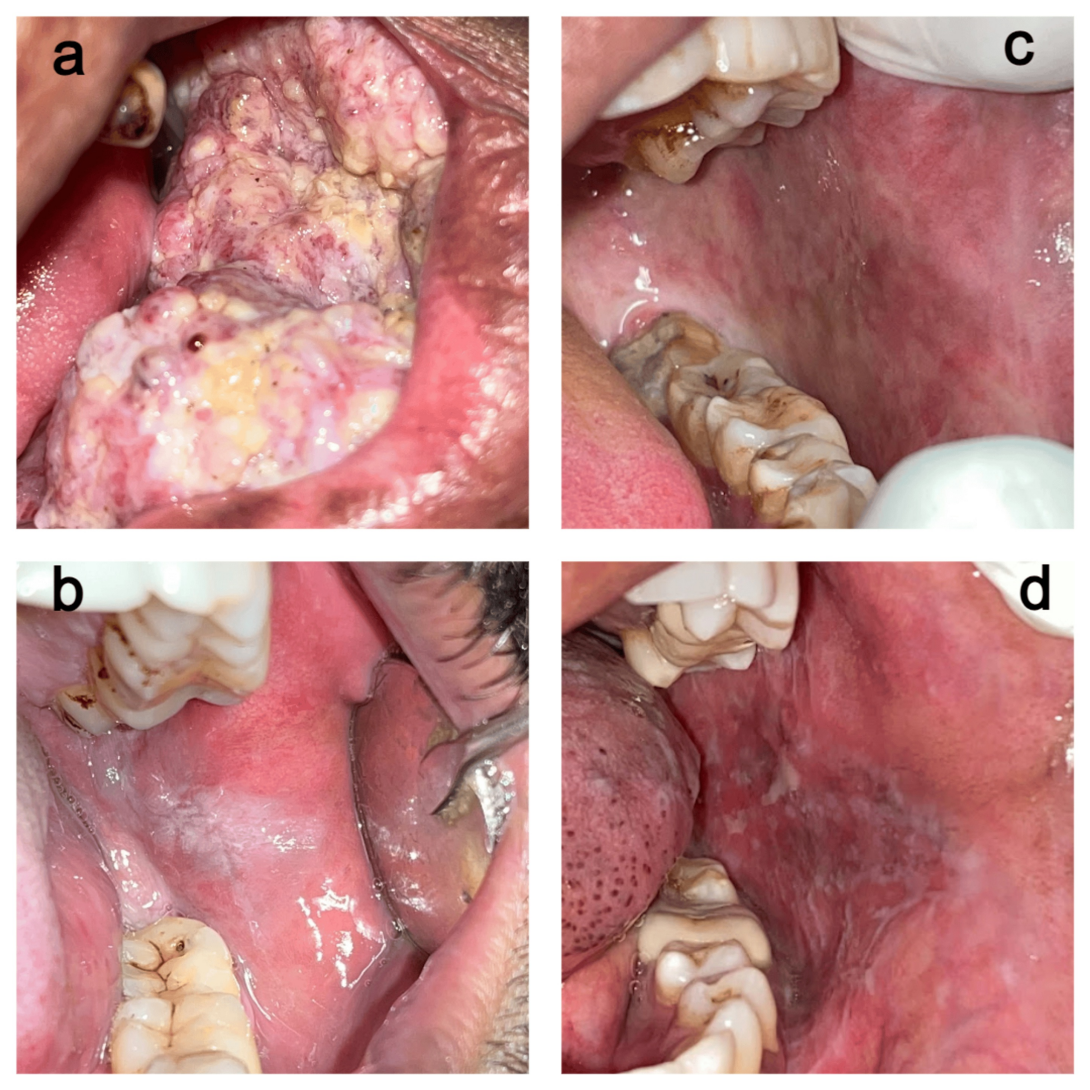
Clinical images of the sample cases included in the study a. Oral cancer, b. Oral leukoplakia, c. Oral submucous fibrosis, d. Lichen planus

The sample size was calculated for a power of 80, using a prior study by Srivastava et al., where the sample size was 14 [16]. To incorporate a 10% loss of attrition it was decided to have 15 samples in each group The study consisted of four study groups: Group 1 (oral cancer): 15 patients, Group 2 (controls): 15 controls, Group 3 (leukoplakia): 15 patients, Group 4 (oral submucous fibrosis + oral lichen planus): 15 patients. The sample images of the group cases are mentioned in Figure 1.

**Figure 2:**
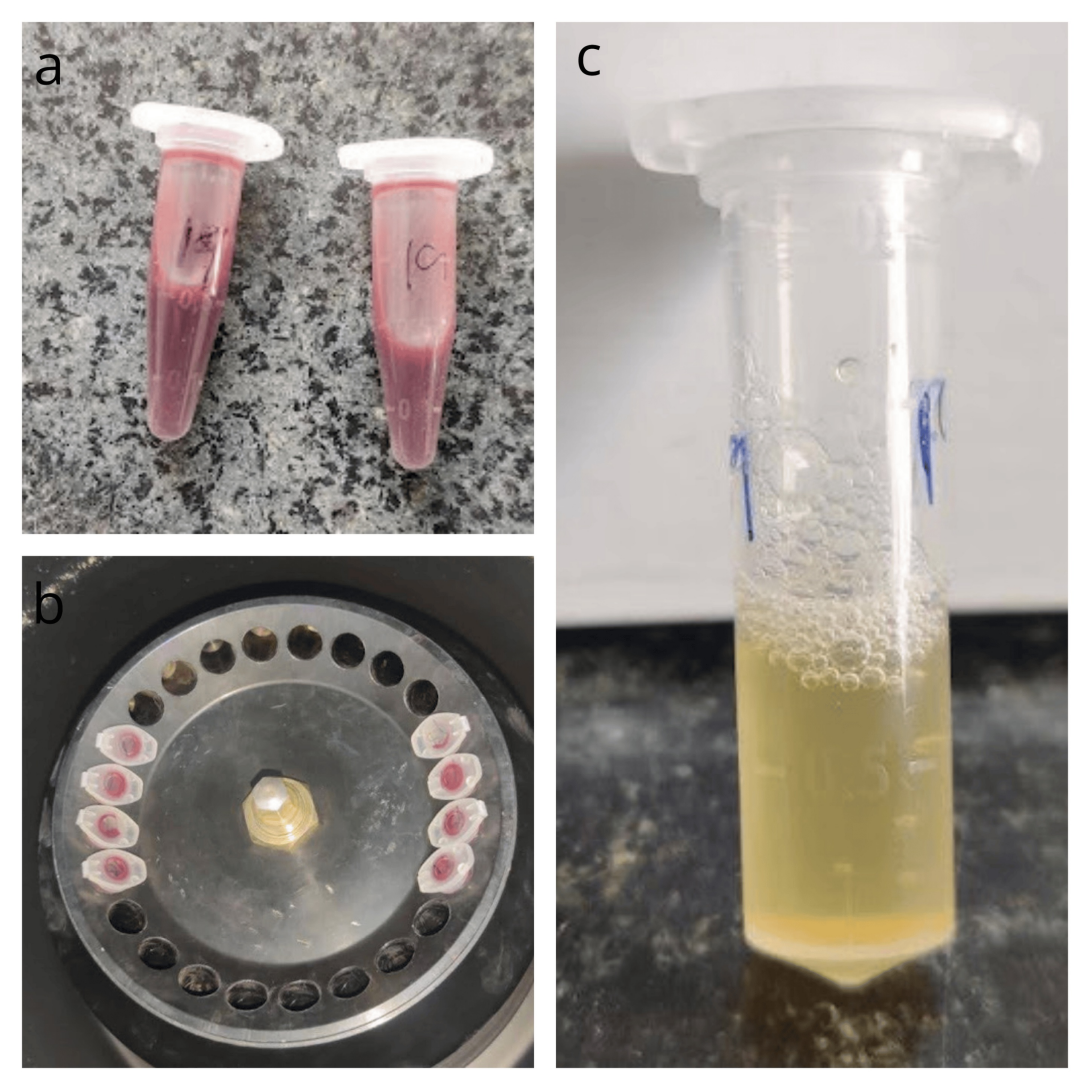
Laboratory processing of the blood samples a. Samples collected in Eppendorf vials, b. Centrifuge, c. Final clear supernatant

The study involved the collection of 5 ml of fresh venous blood from all four groups (Figure 2a). The collected blood was centrifuged at an RPM of 5000 for 5 minutes and in vacutainer vials (Figure 2b). The centrifuged sample showed a clear separation of serum, which was pipetted out and transferred to Eppendorf vials (Figure 2C). This was stored at -80 °C and was analyzed using the LPO ELISA kit.

LPO ELISA kit: methodology 

The study was performed using the Abbkine LPO ELISA kit (Catalogue Number- KTE101140; Atlanta, Georgia, USA). This human LPO ELISA kit was designed for the quantitative determination of LPO levels in various human samples through a two-site sandwich ELISA format. The human LPO ELISA kit comes with a comprehensive set of components with high sensitivity and specificity. The kit includes a pre-coated microplate with specific anti-LPO antibodies, LPO standards for calibration, a detect antibody for LPO, and streptavidin-horse radish peroxidase (HRP) for signal development. Additional materials provided are standard diluent, assay buffer, HRP substrate for the development of the colorimetric signal, a stop solution to halt the reaction, wash buffer for plate washing steps, and plate covers to prevent evaporation and contamination during incubation periods. This LPO ELISA kit should be stored unopened at temperatures ranging from 2-8 °C to ensure its stability and longevity. Before beginning the assay, all reagents should be brought to room temperature for at least 30 minutes. To prevent cross-contamination, fresh pipet tips should be used for each sample. Unused wells need to be desiccated at 4 °C to maintain their integrity. Thorough mixing of the contents is crucial for consistent results, which can be achieved with a low-frequency oscillator or by manual shaking every 10 minutes.

The kit's microplate wells are pre-coated with an antibody specific to LPO, ensuring that only the target enzyme is captured during the assay procedure. To initiate the ELISA, the serum samples were pipetted into the wells and later with the presence of LPO within the samples, it binds to the immobilized antibody. To detect the bound LPO, a biotin-conjugated secondary antibody specific for LPO was added to the wells, forming a bridge between the captured enzyme and the detection system. Following the binding step, the plate undergoes a washout process to remove any unbound biotinylated antibody. The next component added is streptavidin conjugated to HRP, which binds tightly to the biotin on the secondary antibody. A subsequent wash removes any unbound streptavidin-HRP complex. For detecting bound HRP, a colorimetric HRP substrate solution is added. The enzyme-substrate reaction produces a color change that is directly proportional to the amount of LPO present in the initial sample. The reaction is halted by adding a stop solution, stabilizing the color, which is then measured using a microplate reader. This colorimetric method is sensitive and allows precise quantification of LPO concentrations by comparing the absorbance of the samples to a standard curve. The assay is typically completed within a working time of three to five hours.

As alternatives, the kit also acknowledges the existence of other peroxidase enzymes, such as MGC129990, MGC129991, SPO, and salivary peroxidase, which could be measured using similar methodologies tailored to their specific detection.

Statistical analysis was performed using SPSS software version 26.0 (IBM Corp., Armonk, NY, USA). Kolmogorov-Smirnov and Shapiro-Wilks tests were used to analyze the normality of the data distribution and then a comparison of three group means was used.

## Results

The study included a total of 60 patients, of which 15 were controls. There were 18 females and 42 males in the group. The mean age of each group is shown in Table 2.

**Table 2:** Age distribution of the participants in the groups

	Sample Size	Age
Group 1: Oral Cancer	15	37.07 ± 2.10
Group 2: Controls	15	25.93 ± 11.94
Group 3: Oral Leukoplakia	15	29.45 ± 6.66
Group 4: OSMF + Oral Lichen Planus	15	26.93 ± 5.93

The normality, Kolmogorov-Smirnov, and Shapiro-Wilks test results revealed that LPO levels did not follow normal distribution. Therefore, to analyze the data, a non-parametric method was applied. To compare LPO values between groups, the Kruskal-Wallis test was used. To analyze the data, SPSS was used. The significance level was fixed at 5% (α = 0.05).

**Figure 3:**
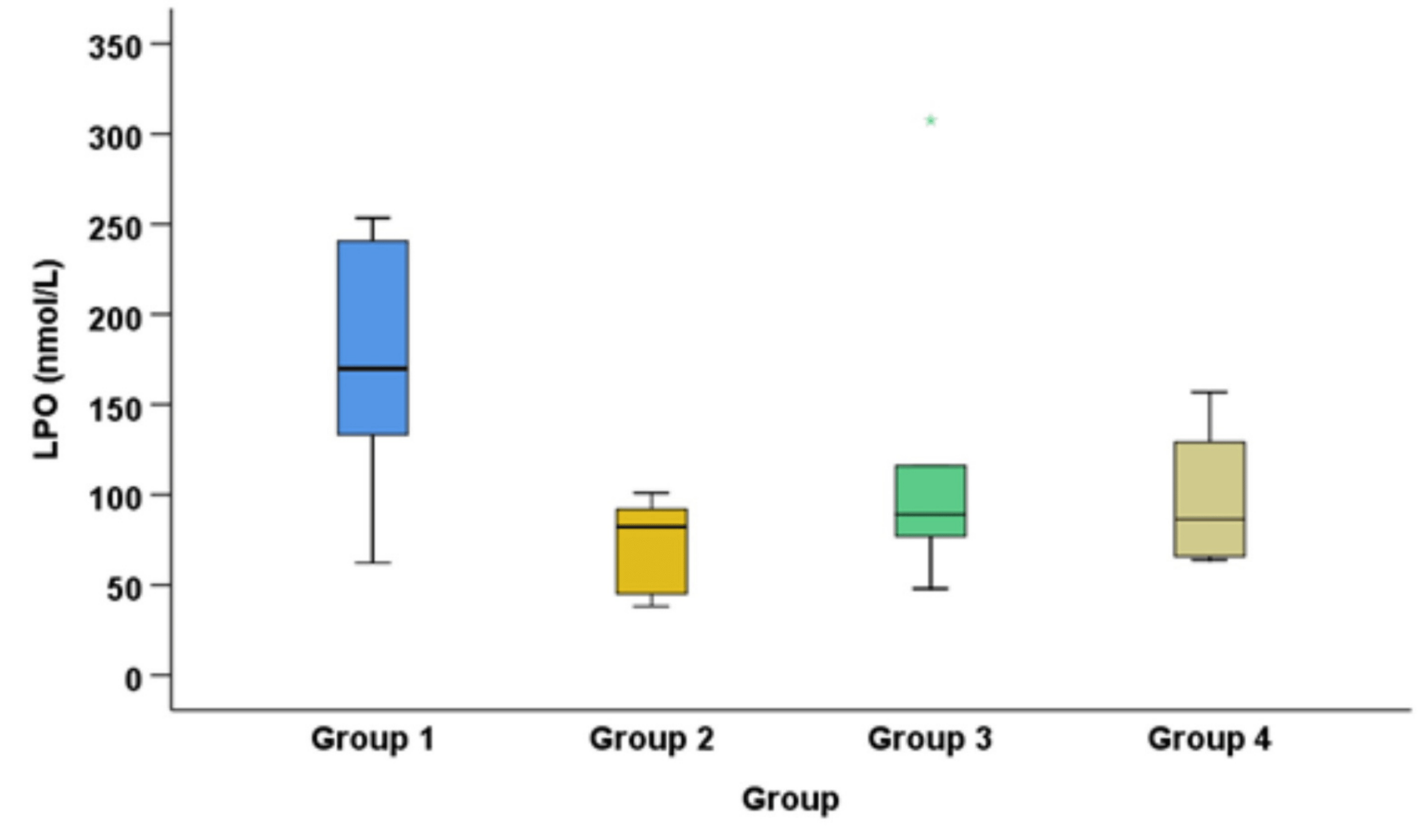
Comparison of lipid peroxidase level between the oral cancer, control, oral leukoplakia, and OSMF + oral lichen planus groups OSMF: oral submucous fibrosis

The results yielded statistically significant differences between the control group and the disease groups (Table 3, Figure 3).

**Table 3:** Descriptive and inferential statistics of lipid peroxidase levels between the groups Group 1: Oral Cancer; Group 2: Controls; Group 3: Oral Leukoplakia; Group 4: OSMF + Oral Lichen Planus OSMF: oral submucous fibrosis

	Lipid peroxidase levels (nmol/L)			
	Group 1	Group 2	Group 3	Group 4
N	15	15	15	15
Median	169.88	82.20	89.10	86.43
1st Quartile	133.18	45.02	76.86	65.73
3rd Quartile	240.63	91.92	116.08	129.10
Mean	171.86	71.66	127.50	100.39
Std Dev	78.86	28.36	103.53	41.06
p-value	0.02			

As a next-level assessment, we decided to check if there was a significant difference between the three disease groups. Though there was a difference in the mean and median, the results were not statistically significant (Table 4).

**Table 4:** Comparison of Lipid Peroxidase levels between the three disease groups Group 1: Oral Cancer; Group 3: Oral Leukoplakia; Group 4: OSMF + Oral Lichen Planus OSMF: oral submucous fibrosis

	Lipid Peroxidase Levels (nmol/L)		
	Group 1	Group 3	Group 4
N	15	15	15
Median	169.88	89.10	86.43
1st Quartile	133.18	76.86	65.73
3rd Quartile	240.63	116.08	129.10
Mean	171.86	127.50	100.39
Std Dev	78.86	103.53	41.06
p-value	0.0543		

## Discussion

The occurrence of OPMD precedes oral cancer. It is essential to identify markers in oral cancer and OPMD, which potentially increase the risk of malignant transformation. ROS has been decisively shown to contribute to the development of malignancy. Lipid peroxidation is the initial event occurring in the mechanism of malignant transformation due to its high cytotoxicity and ability to block protective enzymes. ROS cannot be directly measured; hence, markers of the effects of ROS have been studied. Lipid peroxidation by ROS leads to the formation of hydroxyl ions, which can form adducts with DNA and cause cell disruption. Hence, measuring ROS activity has been a practice for the byproducts of lipid peroxidation. One byproduct of lipid peroxidation, malondialdehyde(MAD), is acknowledged as a co-carcinogenic agent [17]. Souza et al. [18] showed that the byproducts of lipid peroxidations are increased in OPMD.

In recent years, the trend has shifted to measuring the enzymes involved in lipid peroxidation, which are altered by ROS, to proactively measure the amount of cellular damage that the byproducts could cause. In a study by Srivastava et al., the levels of the antioxidant enzyme superoxide dismutase were 14.28±0.67 and 18.54±0.54 in oral cancer and normal patients, respectively. The lower level of anti-oxidant enzyme in oral cancer is suggestive that there was a higher usage of the enzyme in oral cancer [19]. However, this study did not include OPMD.

Studies by Naeem et al. and Shahi et al. assessed the lipid peroxidase in oral submucous fibrosis and oral potentially malignant conditions, respectively. The results revealed that there was a significantly higher level of lipid peroxidation observed in oral submucous fibrosis than that of the healthy controls [20,21]. In the study by Naeem et al., it was noticed that in OSMF the level of malondialdehyde was 3.22 ± 1.265 compared to 1.26 ± 0.568 in controls [20]. Similarly, there was a significant decrease in the enzymatic and nonenzymatic antioxidant levels in precancerous lesions, implying a higher oxidative stress in the study by Shahi et al. These results were on par with our current study, where there was a significant upregulation in the lipid peroxidase levels [21]. A study by Metgud et al. assessed the serum and salivary lipid peroxidation in patients with oral leukoplakia and oral cancer and compared them with that of the healthy controls. The results of the study showed the levels of malonialdehyde as 2.93 ± 0.79 in controls, in leukoplakia as 2.93 ± 0.79, and in oral cancer as 6.02 ± 0.43. The elevation of one of the byproducts of peroxidation was found to be elevated in oral cancer compared to OPMDs and controls [22]. The results are in line with the observations in our study.

The measurement of LPO in OPMD of serum, saliva, and tissue samples was carried out by Ganesan et al. The rise of LPO in OPMD was higher and very consistent in serum and tissue rather than in saliva. The likely reason is that protein degradation in saliva is a little faster [23]. Anti-oxidant markers like glutathione, malondialdehyde, and superoxide dismutase play a crucial role not only in oral potentially malignant disorders but also in other dental conditions like chronic periodontitis. A study by Lopez et al. proves that there were increased antioxidant level markers, such as superoxide dismutase and glutathione peroxidase, in patients with diabetes mellitus who are associated with significant chronic periodontitis [24]. Similarly, studies by Biju et al. proved the association of increased antioxidant levels with patients presenting with periodontitis [25].

The limitations of the current study are that it is a single-point observational study. There was no long-term follow-up and the change in LPO levels with therapy was not monitored. Another major limitation of the study was that the level of LPO according to the grading of oral cancer was not done.

## Conclusions

The current observational study reveals a significant elevation of LPO in the disease groups as compared to the normal. However, there is no significant difference between the disease groups. From this study, it is suggested that LPO could be used as a marker and as a screening tool to assess the rate and severity of cellular damage in patients with potentially oral malignant disorders. This also indicates the amount of dysplastic change. However, a longitudinal follow-up study would help us analyze the change in levels with the disease treatment.
